# Comparative Assessment of TSPO Modulators on Electroencephalogram Activity and Exploratory Behavior

**DOI:** 10.3389/fphar.2022.750554

**Published:** 2022-04-04

**Authors:** Rochelle M. Hines, Elaine A. Aquino, Matthew I. Khumnark, Maria P. Dávila, Dustin J. Hines

**Affiliations:** Department of Psychology, Psychological and Brain Sciences & Interdisciplinary Neuroscience Programs, University of Nevada, Las Vegas, Las Vegas, NV, United States

**Keywords:** TSPO (18 kda translocator protein), PK11195, Ro5-4864, XBD-173, electroencephalography (EEG), open field test (OFT)

## Abstract

Network communication in the CNS relies upon multiple neuronal and glial signaling pathways. In addition to synaptic transmission, other organelles such as mitochondria play roles in cellular signaling. One highly conserved mitochondrial signaling mechanism involves the 18 kDa translocator protein (TSPO) of the outer mitochondrial membrane. Originally, TSPO was identified as a binding site for benzodiazepines in the periphery. It was later discovered that TSPO is found in mitochondria, including in CNS cells. TSPO is implicated in multiple cellular processes, including the translocation of cholesterol and steroidogenesis, porphyrin transport, cellular responses to stress, inflammation, and tumor progression. Yet the impacts of modulating TSPO signaling on network activity and behavioral performance have not been characterized. In the present study, we assessed the effects of TSPO modulators PK11195, Ro5-4864, and XBD-173 via electroencephalography (EEG) and the open field test (OFT) at low to moderate doses. Cortical EEG recordings revealed increased power in the δ and θ frequency bands after administration of each of the three modulators, as well as compound- and dose-specific changes in α and γ. Behaviorally, these compounds reduced locomotor activity in the OFT in a dose-dependent manner, with XBD-173 having the subtlest behavioral effects while still strongly modulating the EEG. These findings indicate that TSPO modulators, despite their diversity, exert similar effects on the EEG while displaying a range of sedative/hypnotic effects at moderate to high doses. These findings bring us one step closer to understanding the functions of TSPO in the brain and as a target in CNS disease.

## Introduction

Neuronal communication predominately occurs through the release of neurotransmitters but many other signaling mechanisms exist in the periphery, and for organisms with a simple nervous system, that relate to metabolism and energy demands. One highly conserved signaling mechanism found in plants, bacteria, and animals centers around translocator protein (TSPO), a small protein that resides on the outer mitochondrial membrane (OMM; (Veenman and Gavish 2006; Li XB. et al., 2015). TSPO was first identified as the Peripheral Benzodiazepine Receptor (PBR) because it acts as a secondary binding site for benzodiazepines, behind the centrally enriched GABA_A_ (γ-aminobutyric acid type A) receptor. TSPO has since been implicated in a multitude of cellular processes and is expressed in the CNS, prompting the name change (Papadopoulos et al., 2006). Although the subject of some recent debate (Selvaraj et al., 2015; Papadopoulos et al., 2018; Betlazar et al., 2020), the most well-known role for TSPO is the transport of cholesterol from the outer to the inner mitochondrial membrane, the rate-limiting step in the production of neurosteroids (Papadopoulos, 1998; Vallée et al., 2001). Other roles indicated for TSPO include porphyrin transport (Verma et al., 1987; Wendler et al., 2003), cellular responses to stress (Batoko et al., 2015), inflammation (Maeda et al., 2007; Rupprecht et al., 2010), and tumor progression (Hardwick et al., 1999; Wu and Gallo 2013). Many of these functions have been defined outside of the CNS, and the role TSPO signaling plays in the brain, particularly how TSPO signaling impacts neuronal network activity is not well understood.

**FIGURE 1:**
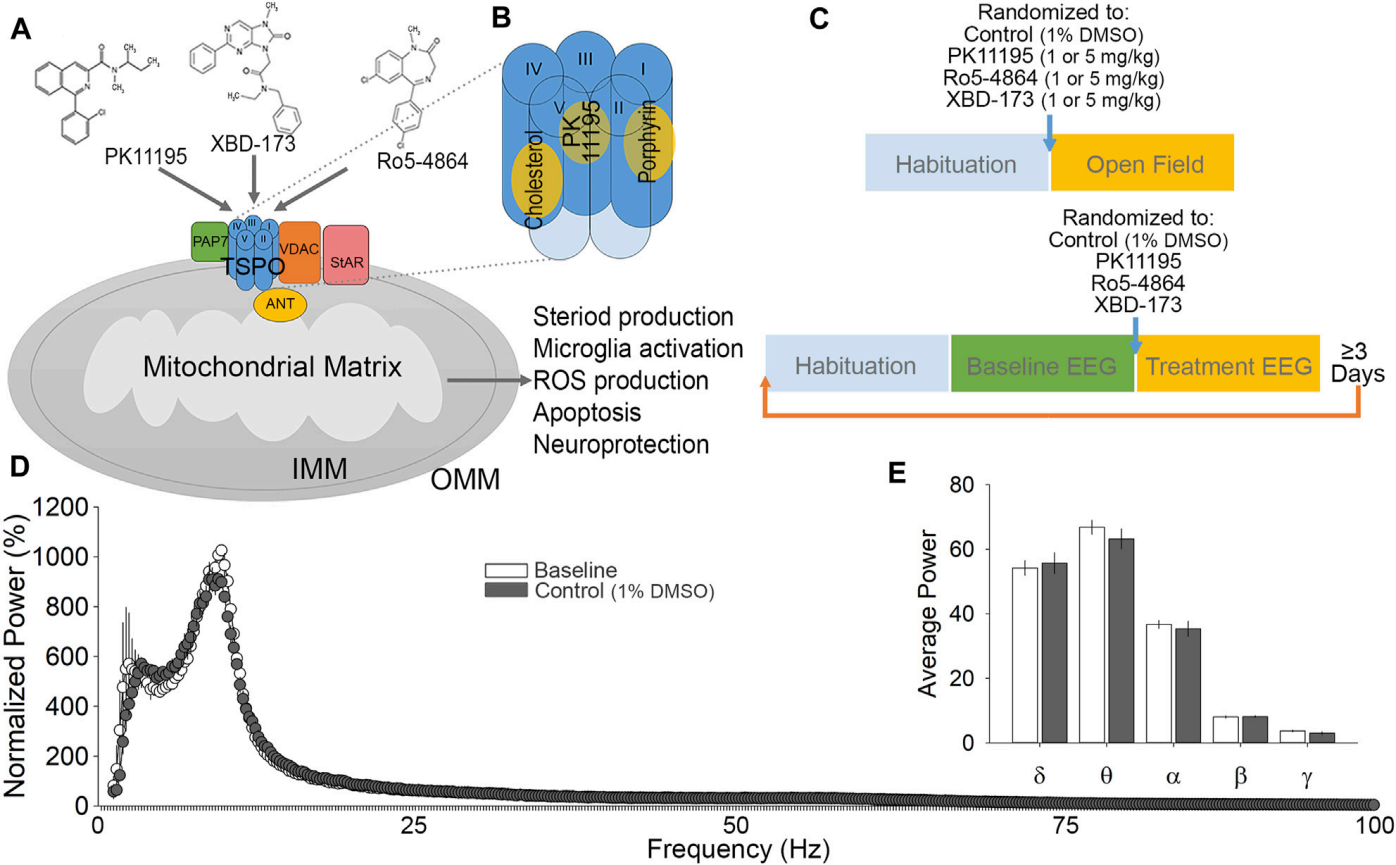
Ligand considerations, experimental design and validation. **(A)** A diagram of TSPO and associated proteins in the outer mitochondrial membrane. TSPO consists of five transmembrane helices and is associated with voltage-dependent anion channel (VDAC), adenine nucleotide transporter (ANT) and TSPO associated protein -7 (PAP7). Binding of PK11195, XBD-173, and Ro5-4864 to TSPO have been demonstrated to alter multiple cellular functions, primarily with regards to endocrine signaling and inflammation. **(B)** Proposed binding pockets for TSPO ligands. **(C)** Experimental timeline for open field test (top) and EEG (bottom) experimental protocols. Each block represents 1 h for habituation (to the testing room for open field; to the recording chamber and tether for EEG), and various phases of assessment (open field behavior, baseline EEG, and treatment EEG). **(D)** Cumulative fast Fourier transform (cFFT) analysis comparing 1-h baseline recording to 1-h following control (1% DMSO vehicle) injection. **(E)** Spectral analysis comparing baseline to control injection. No significant difference was detected in any comparison between baseline and control injection EEG recordings.

Within the brain, TSPO is enriched in the outer mitochondrial membrane, particularly within microglia and endothelial cells (Figure 1A; (Zhang et al., 2014)). X-ray crystallography and nuclear magnetic resonance have revealed the pentameric structure of TSPO, indicating multiple selective binding sites (Figure 1B; (Korkhov et al., 2010; Jaremko et al., 2014, 2015; Li F. et al., 2015). TSPO associates with other mitochondrial proteins such as voltage-dependent anion channel (VDAC), TSPO-associated protein 7 (PAP7), and adenine nucleotide transporter (ANT; Figure 1A; (Soustiel et al., 2008; Miller 2013)). Ligands for TSPO are best known as neuroinflammation biomarkers, in a wide variety of neurodegenerative disorders, including traumatic brain injury and Alzheimer’s disease (Gulyás et al., 2011; Donat et al., 2017; Bauckneht et al., 2019; Werry et al., 2019; Zhang and Gao 2021). In disease states, TSPO becomes highly expressed in microglia, macrophages, and reactive astrocytes with extent and distribution varying with disease (Maeda et al., 2007; Ji et al., 2008; Cosenza-Nashat et al., 2009; Lavisse et al., 2012). Due to its upregulation in a wide range of pathologies, TSPO ligands are frequently used as biomarkers, with radiolabeled [^11^C]PK11195 having been used for more than 2 decades. PK11195 binds with substantial specificity and affinity to TSPO (Verma and Snyder 1989; Owen et al., 2011), and does not show cross-tolerance with diazepam (File 1984; Weiss et al., 1995), suggesting minimal action at GABA_A_ receptors. Beyond PK11195, many other variants and categories of ligands have been developed (Varley et al., 2015; Kim and Pae 2016; Lacapere et al., 2020). Classes of ligands include isoquinoline carboxamides (PK11195), arylindol acetamides (XBD-173), and benzodiazepines (Ro5-4864; Figure 1A), among numerous others (Rupprecht et al., 2010). Although TSPO ligands are used extensively in PET scanning of neurotrauma and neurodegeneration patient populations, we still do not understand what impacts these ligands may have on normal brain function, and the full extent of therapeutic potential that may exist by modulating TSPO function continues to be revealed (Veenman and Gavish 2000, Veenman and Gavish 2006; Dimitrova-Shumkovska et al., 2020; Veenman 2020; González-Blanco et al., 2021).

TSPO and the binding of its ligands are associated with a broad range of physiological outcomes (Le Fur et al., 1983; Soustiel et al., 2008; Chung et al., 2013; Lee et al., 2016). Because of the lack of clarity in the mechanisms of TSPO function and the biological outcomes of drugs acting on TSPO, current literature terms these as drug ligands or modulators as opposed to classifying them as agonists or antagonists (Costa et al., 2016; González-Blanco et al., 2021). PK11195 is an isoquinoline carboxamide ligand for TSPO, and has been in use since 1986 (Charbonneau et al., 1986). Although ligands with better signal to noise and higher specificity have been developed, PK11195 is a very well characterized compound. The saturation binding or equilibrium Ki of PK11195 is 3.60 ± 0.41 nM, and it has been shown to have a short residence time on TSPO of approximately 33 min (Costa et al., 2016). Ro5-4864, also known as 4′-chlorodiazepam, is a benzodiazepine derivative that does not act as a positive allosteric modulator at GABA_A_ receptors, but has been shown to modulate TSPO function in the nM range (Verma and Snyder 1989). Ro 5-4864 is reported to be anxiogenic (5 mg/kg; (File and Lister 1983)), sedative/hypnotic (20 mg/kg; (File and Pellow 1983)), and convulsant (30 + mg/kg; (File et al., 1984)) in rodents, depending upon the dose used. The proconvulsant action (Pellow and File 1984a) is unexpected given that all of the centrally active benzodiazepines are anticonvulsant (Ochoa and Kilgo 2016). Ro5-4864 has an equilibrium K_i_ of 20.04 ± 2.36 nM, and a short residence time on TSPO of 32 min (Costa et al., 2016). Ro5-4864 is essentially inactive at displacing radiolabeled diazepam from GABA_A_ receptors (IC50 of 163,000 nM), but has a very high affinity for TSPO (IC50 of 4.1 nM; (Braestrup and Squires 1977)). At high doses, Ro5-4864 has been demonstrated to block the Cl-ionophore of GABA_A_ receptors (Pellow and File 1984b), which may be responsible for its proconvulsant effects. PK11195 can reduce the incidence of seizures resulting from Ro5-4864 treatment (Bénavidès et al., 1984), suggesting that these ligands may have opposing effects. XBD-173, also known as AC-5216 or Emapunil, is an arylindol acetamide. XBD-173 is considered an agonist for TSPO and has been demonstrated to be anxiolytic, in addition to being neuroprotective by shifting proinflammatory to anti-inflammatory microglia activation during disease (Rupprecht et al., 2009; Gong et al., 2019). XBD-173 is among the more recently developed TSPO ligands, and has a Ki of 0.297 nM and a relatively long residence time on TSPO of approximately 127 min (Kita et al., 2004), which sets it apart from PK11195 and Ro5-4864 (Costa et al., 2016).

The current study aims to examine the impact of TSPO modulation on neuronal activity and CNS output via electroencephalogram (EEG) recording and behavioral assessment in the open field test (OFT). The OFT was selected because it allows analysis of motor activity, exploratory behavior, habituation to a novel environment, and can also provide indications of the level of anxiety of the animal. EEG recording was selected to measure cortical activity because it is highly translational to humans, and is well established to reflect the behavioral state. In the present study, we use these techniques to examine the impact of representative TSPO ligands from three distinct classes comparing the isoquinoline carboxamide PK11195, the arylindol acetamide XBD-173, and the benzodiazepine Ro5-4864 under non-pathological conditions. Drug dosages were set based on the production of mild behavioral phenotypes previously reported, but non-sedating and well below the threshold for proconvulsant activity (File 1984; Gavioli et al., 2003; Rupprecht et al., 2009). We hypothesized that despite being from distinct classes, these modulators would have similar behavioral and EEG effects, and further that these compounds would all act to reduce motor activity in response to the novel environment of the OFT without obvious sedation. Our findings provide a starting point for examining the dose range, behavioral impacts, and pharmacological utility of these important and diverse compounds.

## Methods

### Animal Care and Maintenance

C57Bl6 mice were group-housed under a 12-h light and dark cycle. Food and water were available ad libitum. All procedures were performed following the Institutional Animal Care and Use Committee (IACUC) guidelines at the University of Nevada, Las Vegas.

### Reagents and Drug Preparation

PK11195 (Tocris), Ro5-4864 (Sigma-Aldrich), and XBD-173 (Sigma-Aldrich) were all prepared in 1% DMSO, 99% saline solution. Intraperitoneal injections (i.p.) were given at a volume of 0.1 ml per 10 g of body weight. The doses of 1 and 5 mg/kg were selected to be lower than prior studies with the tested ligands examining behavioral effects, attempting to fall below the established doses for sedation, and well below the dose needed for proconvulsant activity by Ro5-4864 (30 + mg/kg). Since the ligands do not all share a binding site (Jaremko et al., 2014, Jaremko et al., 2015), we did not attempt to base dosing on K_i_, but rather focused on the behavioral impacts of the ligands.

### Open Field Test

A total of 37 mice were used for the OFT: Control (1% DMSO vehicle) n = 5, PK11195 *n* = 12 (6–1 mg/kg and 6–5 mg/kg), XBD-173 *n* = 11 (6–1 mg/kg and 5–5 mg/kg), and Ro5-4864 *n* = 9 (5–1 mg/kg and 4–5 mg/kg). Mice were handled and given habituating saline injections daily for 5 days before behavioral testing. Behavioral testing was initiated 2–4 h into the dark (waking) phase of the light dark cycle. Before behavioral testing, mice were moved to a quiet test room and given 1 hour to habituate to the room environment (Figure 1C). Mice were then randomized into drug and dosage groups and given i. p. injections before being immediately placed in the center of a 44 × 44 cm x 44 cm opaque plexiglass arena for recording under red light. Video was captured using an overhead HDMI camera with Open Broadcast Software (OBS), for a total recording period of 1 h (Figure 1C). After the recording period, mice were returned to their home cages. Tracking of mice in the OFT was automated using ANY-maze software (Stoelting). X Y paths and heat maps were generated using Matlab (The MathWorks, Inc.). Average speed, distance traveled, and time immobile graphs were generated in Sigmaplot (SysStat Software Inc.).

### Electroencephalogram Surgeries

Anesthesia was induced and maintained using isoflurane. A small incision was made on the scalp exposing the skull. For implantation of EEG electrodes, four insulated wire electrodes were placed and screwed as follows: two extradural cortical electrodes were inserted bilaterally in the frontal areas and the two others were inserted bilaterally in the parietal/occipital areas. For implantation of EMG, two insulated wire electrodes were inserted bilaterally into the nuchal muscle. Electrodes connected to a microconnector (Pinnacle Technology) were secured at the surface of the skull with dental acrylic. Following implantation animals were given a postoperative injection of saline for hydration and housed in single cages. Mice were given 1 week to recover before experimentation. Mice were handled and given habituating saline injections daily for 5 days before EEG/EMG recording began as detailed below.

### Electroencephalogram Recordings

The recording apparatus consisted of a head mount that connected to a pre-amplifier, commutator, digitizer, and a computer with Sirenia Acquisition (Pinnacle Technologies, Lawrence, KS). The Pinnacle EEG/EMG system is engineered to be artifact free, with amplification and filtering at the head by the preamplifier, and further by the data conditioning and acquisition system. Data were acquired with a sampling rate of 1 kHz, low pass 0.1 Hz, high pass 100 Hz. Mice were placed in a clear plexiglass cylinder with cob bedding and the pre-amplifier was plugged-in to the head mount implant for at least 1 h to habituate to the recording apparatus and tether (Figure 1C). Recordings were taken of 1 h of baseline activity, beginning 2–4 h into the dark phase of the light dark cycle (Figure 1C). During baseline recordings, animals were visually monitored under red light and sleep was suppressed with the introduction of novel objects in order to help establish a homogenous waking EEG baseline. Following baseline, mice were administered i. p. injections of either control (1% DMSO vehicle; Figures 1D,E), or PK11195, XBD-173 or Ro5-4864, at 1 or 5 mg/kg. Recordings continued uninterrupted for 1 h after injection (Figure 1C), during which time animals were not observed to sleep based on behavioral observation under red light. A total of n = 8 mice per treatment group were utilized for EEG recordings (32 mice total). Mice were given a minimum of 3 days between dose trials to minimize the effects of the previous trial, and were given the 1 mg/kg dose first. Acquired data was exported from Sirenia Acquisition and processed using Sleep Sign for animal (KISSEI COMTEC CO, Nagano, Japan) to generate cumulative fast Fourier transform (cFFT) data, and to allow epoch screening for artefacts. No movement artefacts, aberrant activity, or seizure-like activity were observed. Wakefulness predominated the baseline recordings, characterized by low-amplitude, moderate to high frequency EEG, and high EMG activity. MATLAB (MATLAB 2019) was used for further analysis of spectral frequencies and to generate spectrograms. Frequency bands were defined as δ (0.4–4.0 Hz), θ (4.5–8.0 Hz), α (8.5–13.0 Hz), β (13.5–30 Hz), and γ (30.5–100.0 Hz) (Drinkenburg et al., 2015). Data were normalized to baseline δ.

### Statistics

All graphs were created using SigmaPlot, with line graphs and bar graphs plotted as mean and standard error. After normality testing (Shapiro-Wilk or Kolmogorov-Smirnov) and testing for equal variance (Brown-Forsyth), analysis was performed using parametric tests including *t*-test, one-way, or repeated measures ANOVA with Bonferroni post hoc analysis when appropriate. Exact *p*-values are shown in figures when the threshold for significance (<0.05) is met, in some cases, “n.s.” denotes non-significant results for clarity.

## Results

### PK11195 Attenuates Motor Behavior and Increases δ and θ Frequency Band Power

**FIGURE 2:**
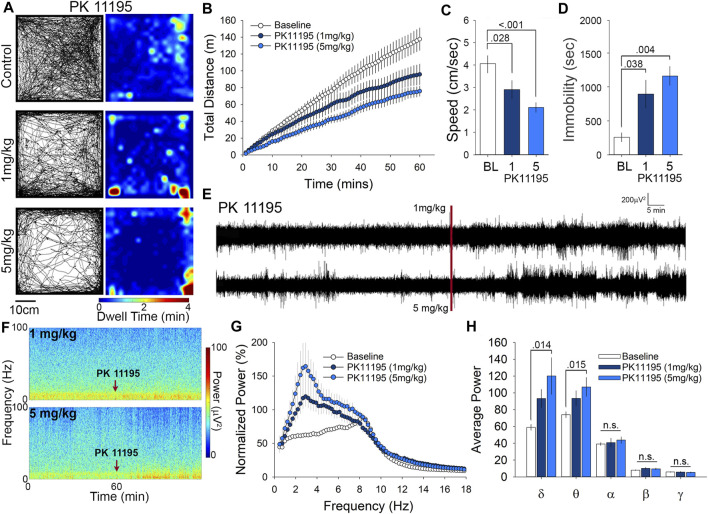
PK11195 reduces locomotion in the open field at both 1 and 5 mg/kg doses and significantly alters δ and θ power at 5 mg/kg. **(A)** Representative paths and heat maps of animal position throughout the open field test. Animals treated with either dose displayed significantly reduced cumulative distance traveled. **(B)** and average speed. **(C). (D)** Treatment with PK11195 also increased time immobile in the open field. **(E)** Raw traces and spectrograms. **(F)** of 2-h EEG recording period spanning baseline and treatment EEG phases. Red line and arrows indicate time of injection. **(G)** Cumulative fast Fourier transform (cFFT) comparing 1-h baseline recordings to recordings following i. p. injection of 1 mg/kg and 5 mg/kg PK11195. **(H)**. Spectral analysis of specific frequency bands in the EEG reveals increases in δ and θ power at 5 mg/kg PK11195.

We administered PK11195 to mice at either 1 or 5 mg/kg and placed them in the OFT for observation of their spontaneous exploratory behavior over 60 min. Animals treated with PK11195 displayed a reduction in overall exploratory behavior as evidenced by the sparser pattern of exploratory paths and long duration stops (yellow to red) in the heat maps of dwell time in the OFT (Figure 2A). To quantify this, we analyzed cumulative distance traveled, and found that this measure was significantly reduced in the 1 mg/kg group by 31% while the 5 mg/kg group had a 50% reduction in cumulative distance traveled (Figure 2B). Average speed was reduced by 29% (1 mg/kg) and 48% (5 mg/kg) (Figure 2C). Relatedly, time immobile was increased in PK11195 treated mice by 2.5 times (1 mg/kg) and 3.5 times (5 mg/kg) (Figure 2D). This examination of OFT activity shows that acute administration of the TSPO ligand PK11195 can elicit reductions in locomotor behavior and that these reductions are more pronounced at higher doses.

Little is known about the effects of PK11195 administration on neuronal circuit activity, so in parallel with the OFT we also examined the effects of PK11195 on EEG. Visual inspection of the raw traces (Figure 2E) and spectrograms (Figure 2F) reveal an elevation in power following injection of both 1 and 5 mg/kg PK11195 compared to baseline. Using fast Fourier transform (FFT) analysis we found that i. p injection of both 1 and 5 mg/kg of PK11195 significantly increased the power of the EEG compared to baseline, particularly in the low frequency bands (Figure 2G). Spectral analysis showed these increases in power to be in slow oscillations (Figure 2F), specifically within the δ (0.4–4 Hz) and θ (4.0–8.0 Hz) range (Figure 2H). There was a 2-fold increase in δ power from baseline, in the 5 mg/kg PK11195 treated animals, and a 50% increase in 1 mg/kg θ power was observed to have an increase of 30% in 5 mg/kg and was not significantly altered by the 1 mg/kg dose. Of note, injection with the 1% DMSO vehicle alone does not significantly alter the EEG power or architecture compared to baseline (Figures 1D,E), demonstrating that the EEG effects of PK11195 are specific to PK11195 and not the vehicle or injection. This analysis of cortical network activity following treatment with PK11195 revealed a band-specific and dose-specific effect.

### Ro5-4864 Attenuates Motor Behavior and Dose Dependently Alters Electroencephalogram Power

**FIGURE 3:**
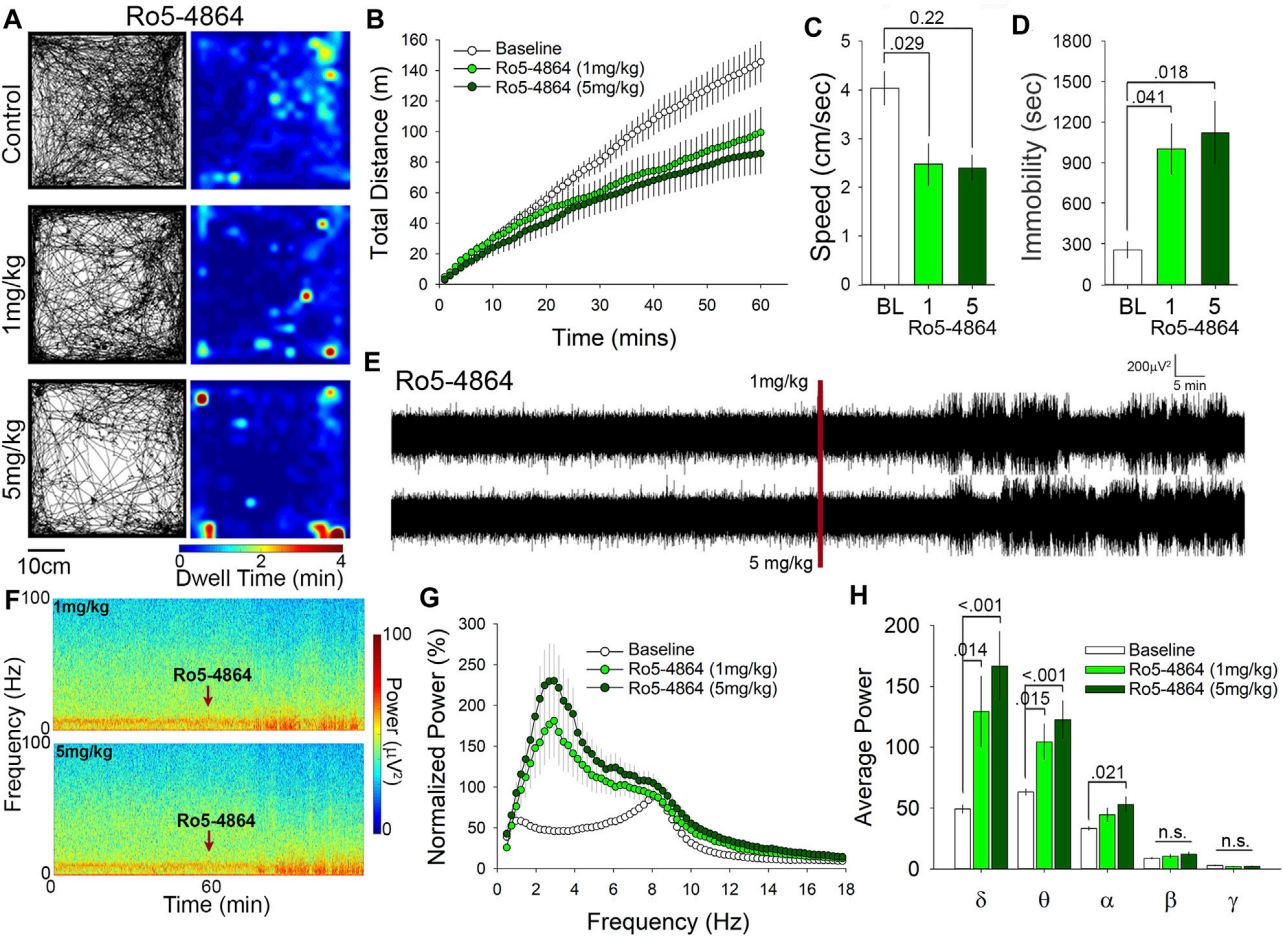
Ro5-4864 reduces locomotion in the open field and increases δ and θ power at both 1 and 5 mg/kg. **(A)** Representative paths and heat maps of animal position throughout the open field. **(B)** Cumulative distance traveled and average speed. **(C)** were significantly reduced in animals treated with both 1 and 5 mg/kg Ro5-4864. **(D)** Treatment with Ro5-4864 also increased time immobile. **(E)** Raw traces and spectrograms **(F)** of 2-h recording period, with the red line and arrows indicating time of i. p. injection. **(G)** cFFT comparing 1-h baseline recordings to recordings following i. p. injection of 1 mg/kg and 5 mg/kg Ro5-4864. **(H)** Spectral analysis of specific frequency bands in the EEG reveals increases in δ and θ power with both 1 and 5 mg/kg Ro5-4864, along with an increase in α power at the higher dose of 5 mg/kg.

We next examined the effects of Ro5-4864 in the OFT at doses of either 1 or 5 mg/kg. Representative paths of exploratory behavior and heat maps of dwell time are suggestive of a reduction in exploratory behavior following both 1 and 5 mg/kg Ro5-4864 compared to control (1% DMSO vehicle injected; Figure 3A). To examine locomotor behavior more closely we quantified the cumulative distance travelled and found that 1 mg/kg of Ro5-4864 resulted in a 35% decrease, while 5 mg/kg produced a 40% decrease compared to control (Figure 3B). Assessment of average speed of travel also suggests reduced locomotor and exploratory behavior at both 1 mg/kg (36% decrease) and 5 mg/kg (41% decrease) of Ro5-4864 compared to control (Figure 3C). Correspondingly, time immobile was increased by Ro5-4864 treatment at 1 mg/kg (4-fold increase) and 5 mg/kg (4.5–fold increase) (Figure 3D). In a prior study, no effect of Ro5-4864 was found in the OFT (Gavioli et al., 2003), however the protocol used was 5 min in the OFT at 1 h following 1 mg/kg treatment with Ro5-4864, as opposed to the 60-min assessment that began immediately following injection in our current experiment. Of particular interest, we found that both PK11195 and Ro5-4864 similarly reduce locomotor and exploratory activity of mice in the OFT despite that some studies suggest they have distinctive mechanisms of action on TSPO (Bénavidès et al., 1984; Caballero et al., 2014).

Administration of Ro5-4864 also produced increases in EEG power that are visible in the representative traces and spectrograms (Figures 3E,F). Similar to PK11195 these increases in power appear to be primarily in the slower wavelengths toward the bottom of the spectrogram (Figure 3F). cFFT analysis again suggested that these changes may be predominantly in the slow frequencies (Figure 3G), which was confirmed by spectral analysis to be specific to the δ and θ frequency bands (Figure 3H). 1 mg/kg Ro5-4864 increased δ power by 2.5-fold and θ power by 1.6-fold, while 5 mg/kg increased δ power by 3.6-fold and θ power by 2-fold (Figure 3H). Although there was no significant difference between 1 mg/kg and 5 mg/kg in δ and θ power, the trend follows the increase in dose. Of interest, we also found a significant increase in α frequency band power at the 5 mg/kg dose of Ro5-4864 (Figure 3H), distinguishing it somewhat from the effects of PK11195.

### XBD-173 Attenuates Motor Behavior at 5 mg/kg and Dose Dependently Alters Electroencephalogram Power

**FIGURE 4:**
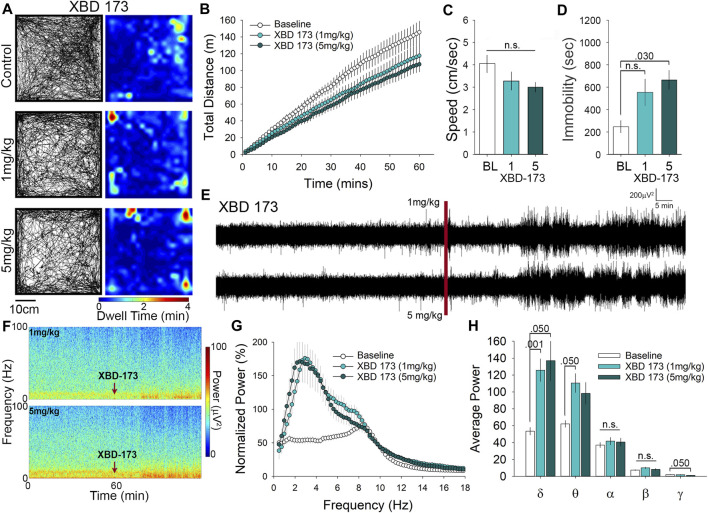
XBD-173 reduces locomotion in the open field at the higher dose of 5 mg/kg yet significantly alters both δ and θ power at 1 mg/kg. **(A)** Representative paths and heatmaps of animal position throughout the open field test. XBD-173 did not result in a significant change in cumulative distance traveled **(B)** or the average speed of travel **(C)** in the open field at either 1 or 5 mg/kg. **(D)** Treatment with 5 mg/kg XBD-173 did significantly increase the time immobile. **(E)** Raw trances and spectrograms **(F)** of the 2-h recording period, with the red line and arrows indicating time of i. p injection. **(G)** cFFT comparing 1-h baseline recordings to recordings following i. p. injection of 1 mg/kg and 5 mg/kg XBD-173. **(H)** Spectral analysis of specific frequency bands in the EEG reveals increases in δ and θ power at 1 mg/kg XBD-173, while at 5 mg/kg δ and γ power are significantly altered compared to baseline.

Our last comparator was XBD-173, which was also administered at either 1 or 5 mg/kg for examination of OFT behavior. Representative paths of exploratory behavior and heatmaps of dwell time in the OFT suggest similar levels of exploration in XBD-173 treated mice compared to controls (Figure 4A). The number of center crossings (path tracings that intersect the center), and the occurrence of stops of short duration (pale blue to turquoise) are relatively consistent with control treated mice (Figure 4A). In order to quantitatively assess locomotor activity, we calculated the cumulative distance traveled (Figure 4B), and also the average speed of travel in the OFT (Figure 4C), which did not differ between the groups. Although there was no significant difference in distance travelled or average speed, we did detect an increase in time immobile of 2.5-fold at the 5 mg/kg dose of XBD-173 (Figure 4D.) Although acute administration of XBD-173 has been reported to have no effect on locomotor behavior (Rupprecht et al., 2009), we found that i. p injection of 5 mg/kg XBD-173 could cause reductions in mobility in the OFT. Locomotor analysis shows XBD-173 to have no significant effect on locomotion and open field behavior at 1 mg/kg.

XBD-173 has been reported to be anxiolytic (Kita et al., 2004; Barron et al., 2021), but there has been little research on the effects of XBD-173 on EEG recordings. Visual inspection of the representative traces and spectrograms suggests that administration of XBD-173 causes an increase in power of low-frequency oscillations similar to that of PK11195 and Ro5-4864 (Figures 4E,F). Of interest, the spectrogram also suggests that there may be further alterations to the EEG induced by XBD-173 in the high frequency range (darker blue near the top of the spectrogram; Figure 4F). cFFT analysis confirmed quantitative increases in the power of the low frequency wavelengths of the EEG following XBD-173 (Figure 4G). To determine which frequency bands were influenced by XBD-173 we performed spectral analysis, which showed that δ power increases by 2.2-fold at 1 mg/kg and 2-fold at 5 mg/kg. There was also a 2-fold increase in θ power that was only found after 1 mg/kg XBD-173, and not with the higher dose. Interestingly, γ power was significantly decreased by 2-fold in the 5 mg/kg dose of XBD-173, but not by 1 mg/kg. These findings reveal a dose-dependent effect on specific frequency wavelengths in the EEG as a result of XBD-173 administration.

### At 1 mg/kg Translocator Protein Ligands Differentially Alter Locomotor Activity in the Open Field Test and Electroencephalogram δ Power

**FIGURE 5:**
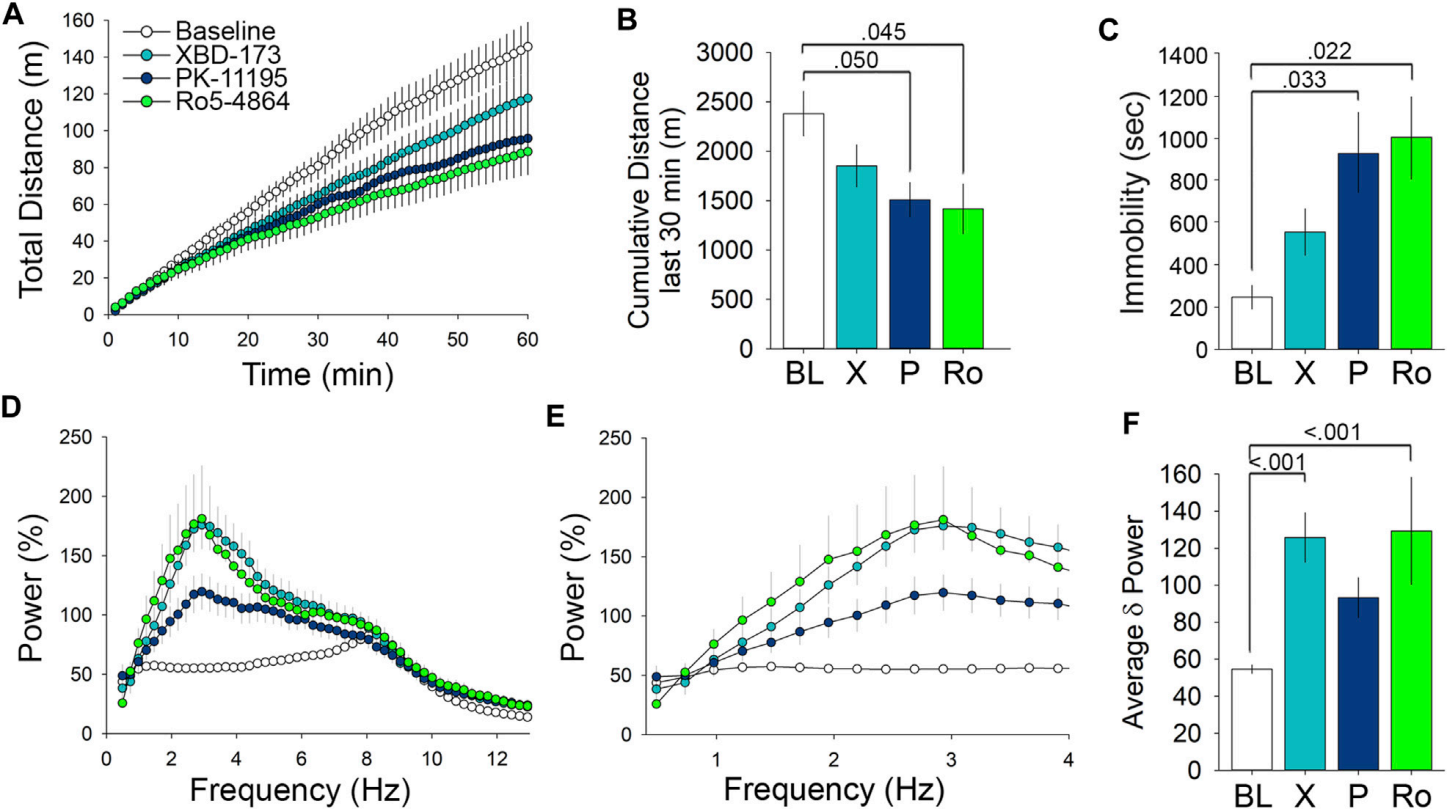
Comparison of the behavioral and EEG effects of TSPO modulators at 1 mg/kg reveals effects on locomotor activity and low frequency δ wavelength power. **(A)** Comparison of the effect of 1 mg/kg doses of the tested TSPO modulators on distance travelled in the open field. **(B)** Measure of the cumulative distance travelled during the last 30 min of the open field comparing the 1 mg/kg doses. **(C)** Immobility in the open field following 1 mg/kg doses. **(D)** cFFT comparing the EEG effects of 1 mg/kg doses of the tested TSPO modulators, showing the prominent effects of XBD-173 and Ro5-4864 on low frequency wavelengths compared to baseline levels. **(E)** cFFT of wavelengths in the δ frequency range comparing the tested TSPO modulators at 1 mg/kg. **(F)** Average δ power showing the elevation caused by 1 mg/kg XBD-173 and Ro5-4864 compared to baseline.

In addition to the individual analysis, we also wanted to compare the effects of the distinctive TSPO ligands. 1 mg/kg dosing of both PK11195 and Ro5-4864 significantly impacted open field behavior. The impact on open field behavior was particularly prominent during the last 30 min of the test, during which both PK11195 and Ro5-4864 both decreased the cumulative distance travelled (Figures 5A,B). While administration of XBD-173 at 1 mg/kg did not significantly alter total time immobile compared to control, both PK11195 and Ro5-4864 significantly decreased time immobile when administered at 1 mg/kg (Figure 5C). This effect is surprising considering that at equivalent doses, both PK11195 and Ro5-4864 should be less effective at modulating TSPO compared to XBD-173 based on K_i_ values. XBD-173 also differs significantly from Ro5-4864 and PK11195 based upon residence time (Kita et al., 2004; Costa et al., 2016), which may be a factor to consider in optimizing the behavioral effects of TSPO modulators for specific clinical indications.

We next performed comparative analysis with the EEG cFFT results, focusing on the low frequency wavelengths (Figure 5D). Narrowing the spectra to 0.4–4 Hz demonstrates the differential effects of the ligands on EEG δ frequency wavelength power (Figure 5E). Spectral analysis of average δ power demonstrated that while both XBD-173 and Ro5-4864 resulted in a significant increase at 1 mg/kg, PK11195 did not (Figure 5F). Again, this is of interest given the relative K_i_ and residence time values of the individual compounds (Kita et al., 2004; Costa et al., 2016). In terms of effects on the EEG, XBD-173 and Ro5-4864 exert similarly pronounced effects despite differences in both K_i_ and residence time (Kita et al., 2004). As shown above, higher doses of all ligands influence EEG δ power. Altogether these results indicate that while XBD-173 caused the least amount of immobility in the OFT, it produced a significant change in EEG δ power. PK11195, on the other hand, caused a significant increase in immobility, but did not significantly change δ power. This information will be useful in continuing to refine TSPO modulators for therapeutic applications.

## Discussion

In these series of experiments, we demonstrate that TSPO modulators from three different classes (PK11195, XBD-173, and Ro5-4864) differentially reduce locomotor activity in the OFT as evidenced by measures of distance traveled, average speed, and time immobile. These effects were found to be both dose-dependent and ligand-dependent. In particular, 1 mg/kg of XBD-173 did not result in reduced locomotion. For XBD-173, a larger dose was needed (5 mg/kg) in order for significant effects on locomotion and exploratory behavior to be observed. In addition to behavioral analysis, we characterized the effects of these ligands on neuronal signaling, via cortical EEG recordings. In these studies, we found broad power increases in δ and θ frequency bands in the hour following injection of each of the TSPO modulators. The increases in the power of the slow frequency bands were dose-dependent, particularly with PK11195 requiring the higher 5 mg/kg dose to increase δ and θ power. We also found ligand and dose-specific changes in power, including increases in α power that were specific to Ro5-4864 at 5 mg/kg, and decreases in γ power that were specific to XBD-173 at 5 mg/kg.

Locomotor tasks such as the OFT and rotarod have previously demonstrated that PK11195 and Ro5-4864 do not impair locomotion (Gavioli et al., 2003). Over a 60 min period, we observed changes in locomotion following administration of both PK11195 and Ro5-4864 at both 1 and 5 mg/kg. In the Gavioli et al. (2003) study they examined 5 min of open field behavior 60 min after the injection. The first 5 min in the open field results in a hyperlocomotor response due to the novelty of the environment, which would lead to masking of the effects of the compounds. Animals need 15 or more minutes to habituate to the open field upon the first exposure requiring a longer testing time. The most pronounced effects of the ligands in our study are seen after 30 min. With a longer OFT protocol, we did not find significant effects on locomotion with the 1 mg/kg dose of XBD-173, while both PK1195 and Ro5-4864 reduced locomotor behavior at both 1 and 5 mg/kg. The low K_i_ value and long residence time of XBD-173 (Kita et al., 2004) may contribute to its differential effects on behavior.

Interestingly, both XBD-173 and Ro5-4864 had similarly robust effects on the EEG, while the effect of PK11195 is less pronounced. The increases in δ and θ power have not been previously observed with Ro5-4864, PK11195, or XBD-173, as there has not been a comprehensive EEG study of these compounds. Limitations of the present study include comparison of EEG effects to baseline EEG, rather than the use of vehicle control treated mice matched with each TSPO ligand administered. There is however benefit to within animal controls, where the resistance and impedance from mouse to mouse can vary. Comparison of vehicle control treatment did not induce any changes in the EEG compared to baseline. Increases in the δ and θ frequency bands have been associated with NREM and REM sleep (Lancel 1993; Gottesmann 1996; McCormick et al., 1997). In particular, the transition to slower frequencies is associated with the transition to sleep, yet natural sleep was not observation in the 60 min following TSPO ligand administration. These findings suggest that ligands modulating TSPO function could be useful in altering sleep architecture without true sedation. Benzodiazepines acting as positive allosteric modulators at GABA_A_ receptors are sedative, but are known to reduce δ and θ power suppressing slow wave sleep, resulting in poor quality of sleep (Aeschbach et al., 1994; Lancel and Steiger 1999). Ro5-4864 is classed as a benzodiazepine, but increased δ and θ power, further confirming its distinctive mechanism of action from classical benzodiazepines. XBD-173 is known to be anxiolytic and could prove to be an alternative to benzodiazepines for the treatment of anxiety disorders.

While the actions of TSPO ligands on EEG and behavior are distinct from benzodiazepines, they do share similarities with the actions of gaboxadol (THIP), a superagonist of GABA_A_ receptors containing the δ-subunit (Krogsgaard-Larsen et al., 2004; Meera et al., 2011). For example, gaboxadol induces immobility in the open field (7 mg/kg; (Bowen et al., 2015)), and enhances EEG δ frequency band power (6 mg/kg; (van Lier et al., 2004; Winsky-Sommerer et al., 2007; Grotell et al., 2021). Intriguingly δ-subunit containing GABA_A_ receptors are also sensitive to physiological levels of neurosteroids (Stell et al., 2003; MacKenzie and Maguire, 2013). The time course of effects observed with the TSPO ligands under study (∼15 min following injection), appears too rapid for enhanced neurosteroid production and secondary action on δ-subunit containing GABA_A_ receptors. Administration of PK11195 (21 mg/kg) in rats has been shown to increase the plasma concentrations of neuroactive steroids after 30 min (Serra et al., 1999). As a further point of comparison, alcohol is known to enhance neurosteroid production and modulate δ-subunit containing receptors, with allopregnanolone being significantly increased approximately 40 min following administration (VanDoren et al., 2000). Thus, despite similarity to gaboxadol it is unlikely that the acute effects of TSPO ligands on EEG and behavior that we observe are mediated by enhanced neurosteroid production. Related to the diverse roles of TSPO, TSPO ligands exert numerous downstream effects (Rupprecht et al., 2021), and further study is required to determine the precise mechanism of the effects we observe.

With respect to the neuroprotective effects of TSPO ligands (Soustiel et al., 2008), the qualities of sedation and slowing of the EEG detected in these studies may contribute to therapeutic benefit. For example, sedative agents are a common tool used in the management of brain injury including cardiac arrest and traumatic brain injury (Flower and Hellings, 2012; Dell’Anna et al., 2014; Oddo et al., 2016; Paul et al., 2018). Traumatic brain injury is associated with changes in the EEG oscillatory activity, and low frequency stimulation to shift the EEG has been shown to improve cognitive outcome in models of traumatic brain injury (Pevzner et al., 2016). A screening strategy examining the spectrum of reduced locomotion to sedation along with slowing of the EEG could help to optimize TSPO ligands for neuroprotection and therapeutic potential.

Taken together, our results suggest that TSPO ligands PK11195, Ro5-4864, and XBD-173 can alter locomotor behavior, and modulate band-specific changes in cortical EEG. Further research needs to be done on the locomotor effects of these ligands, as well as their potential effects on sleep architecture. Benzodiazepines can bind TSPO, and TSPO selective ligands have similar yet distinct effects on neuronal circuits and behavior from those of benzodiazepines, suggesting complementarity. These unique effects make TSPO an alluring target for the development of novel pharmaceuticals for psychiatric disorders such as anxiety, as well as sleep disorders.

## Data Availability

The raw data supporting the conclusions of this article will be made available by the authors, without undue reservation.
